# Histon activities in the extracellular environment: regulation and prothrombotic implications

**DOI:** 10.1097/MOH.0000000000000827

**Published:** 2024-05-27

**Authors:** Gwen M. Keulen, Joram Huckriede, Kanin Wichapong, Gerry A.F. Nicolaes

**Affiliations:** Department of Biochemistry, Cardiovascular Research Institute Maastricht (CARIM), University of Maastricht, Maastricht, The Netherlands

**Keywords:** extracellular histones, immunothrombosis, NETosis, thromboembolism, thromboinflammation

## Abstract

**Purpose of review:**

Thromboembolic complications are a major contributor to global mortality. The relationship between inflammation and coagulation pathways has become an emerging research topic where the role of the innate immune response, and specifically neutrophils in “immunothrombosis” are receiving much attention. This review aims to dissect the intricate interplay between histones (from neutrophils or cellular damage) and the haemostatic pathway, and to explore mechanisms that may counteract the potentially procoagulant effects of those histones that have escaped their nuclear localization.

**Recent findings:**

Extracellular histones exert procoagulant effects via endothelial damage, platelet activation, and direct interaction with coagulation proteins. Neutralization of histone activities can be achieved by complexation with physiological molecules, through pharmacological compounds, or via proteolytic degradation. Details of neutralization of extracellular histones are still being studied.

**Summary:**

Leveraging the understanding of extracellular histone neutralization will pave the way for development of novel pharmacological interventions to treat and prevent complications, including thromboembolism, in patients in whom extracellular histones contribute to their overall clinical status.

## INTRODUCTION

Thromboembolic complications are a major contributor to global mortality [1]. Over the years, many causal factors and mechanisms have been put forward that contribute to the aetiology of thrombotic disease. In recent years new insights have highlighted a special role that inflammatory responses may play in the onset and propagation of thromboembolic processes. Understanding the intricate relationship between inflammation and coagulation pathways has become a major research focus. In this respect “thromboinflammation” and “immunothrombosis” specifically focus on the interplay between the immune system and thrombotic disease [2–4].

Immunothrombosis may be instigated in both sterile (cellular damage) and nonsterile (invasion of pathogen) inflammation [5–8]. During inflammation, neutrophils play a pivotal role by the formation of neutrophil extracellular traps (NETs). Although also other cell types have been reported to contribute to extracellular trap (ET) formation [9], due to their abundance in human blood, neutrophils are regarded as very important to ET formation [10]. NETs serve to ensnare and eliminate invading pathogens at infection sites, and are comprised of a complex matrix of DNA, histones, myeloperoxidase (MPO), neutrophil elastase (NE), and antimicrobial granules [11]. Histones are an integral and essential part of NETs, where they weaponize the extracellular traps by bringing toxic properties that contribute to their antipathogenic activities [12,13]. Being cytotoxic, extracellular histones are indiscriminate and can cause damage to host cells [14]. In particular, extracellular histone H3 and histone H4 can bind to cellular membranes as well as trigger pattern recognition receptors (PRRs). The endothelial inner lining of blood vessels is particularly susceptible to histones and it is here that a potential vicious circle is triggered: extracellular histones induce endothelial cell death, which, if these cells are not properly cleared, may result in release of additional histones from the dying endothelial cells [15]. This cycle can repeat and amplify itself and is thought to contribute to such fulminant diseases as sepsis or other systemic inflammatory conditions which are known to be associated with thrombotic conditions, like diffuse intravascular coagulation (DIC) [2]. 

**Box 1:**
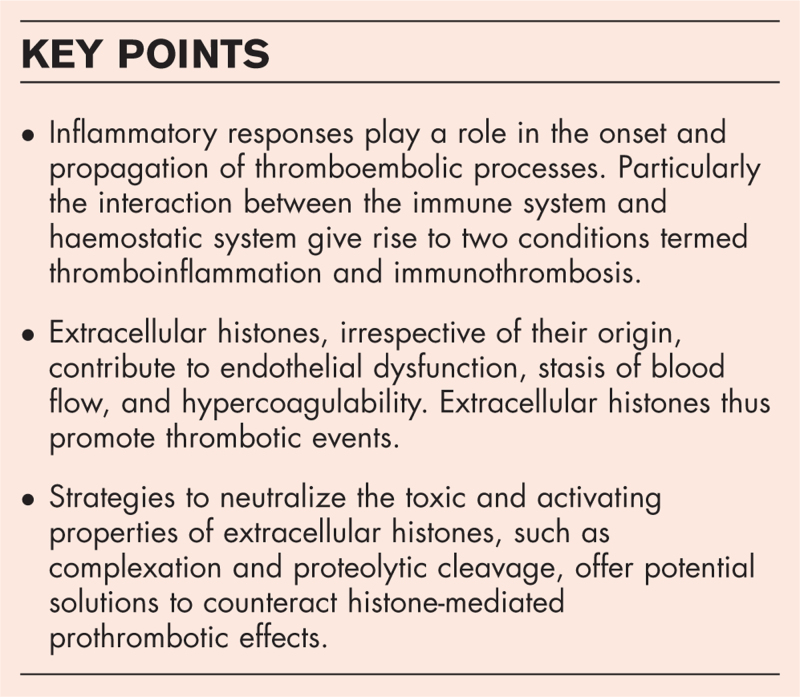
no caption available

**FIGURE 1:**
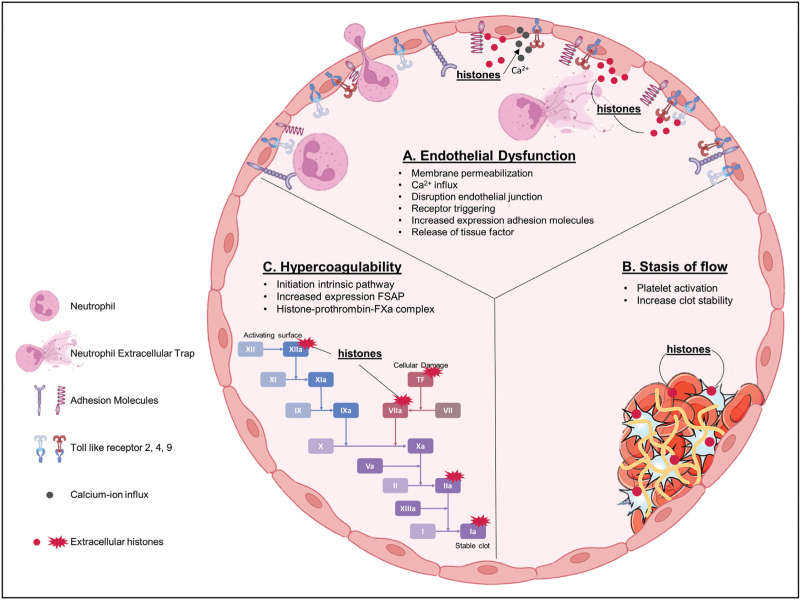
The effect of extracellular histones on the elements of the Virchow triad. (a) Extracellular histones induce endothelial dysfunction by increased expression of adhesion molecules, damage of cell junctions, tissue factor release and cytotoxicity to endothelial cells via direct (membrane permeabilization and calcium influx) and receptor mediated mechanisms (TLR 2, TLR 4, TLR 9, NOD-Like receptors). (b) Histones increase platelet activation, adhesion, and aggregation during primary haemostasis. Fibrin fibre thickness and density of network are influenced by histones. These effects stabilize the clot structure and impair the blood flow through the vessel. (c) Extracellular histones interact with coagulation proteins at different stages of the coagulation pathway. All effects lead to a pro-coagulant phenotype.

Histones are capable of activating many cell types, which in response can become prothrombogenic by providing essential surfaces on which coagulation may occur. Other, more direct mechanisms exist by which histones can tip the haemostatic balance as detailed below [16^▪▪^]. Direct interaction of histones with coagulation proteins such as FXa, FVII activating protease (FSAP), and prothrombin as well as activation of platelets contribute to the histone-mediated hypercoagulability [16^▪▪^]. In a more classical view, extracellular histones contribute to all three factors of Virchow's triad: endothelial damage, stasis of flow, hypercoagulable state; which collectively can lead to thromboembolic events (Fig. 1). In light of this, interventions targeting extracellular histones have emerged as a promising strategy to mitigate immune-driven thrombotic processes [3]. This review aims to dissect the intricate interplay between histones and the haemostatic pathway, and to explore mechanisms that may counteract the potentially procoagulant effects of those histones that have escaped their nuclear localization.

## THE EFFECT OF EXTRACELLULAR HISTONES ON THE ELEMENTS OF THE VIRCHOW TRIAD

Following the classical view by Virchow on thrombosis, it is possible to provide an insight into the multifaceted mechanisms by which histones can contribute to the initiation and propagation of thrombotic disease. The following sections provide an insight into how endothelial injury, stasis and hypercoagulability are influenced by the presence of extracellular histones, that are able to tip the haemostatic balance towards a more prothrombotic state.

### Histone-mediated endothelial activation and dysfunction

Endothelial injury plays an important role in the formation of thrombi, mediated through several factors, including the loss of protective molecules, the expression of adhesive molecules, and by providing a procoagulant surface. Healthy endothelium is decorated with a protective carbohydrate-rich layer known as the glycocalyx, to preserve adequate tissue perfusion [11,16^▪▪^,^17^,^18^]. Damage to the glycocalyx and endothelial cells exposes adhesion molecules and the subendothelial matrices, enhances permeability, and activates coagulation [19–21] (Fig. 1a).

Histones can exert such damage and activation through direct and receptor-mediated mechanisms. Examples of direct mechanisms are permeabilization of the plasma membrane and mitochondrial membranes, ionophore formation with subsequent calcium influx, and disruption of the junctional continuity between endothelial cells [17,22–25]. Studies employing HUVECs treated with histones demonstrate impairment of endothelial junctions [16^▪▪^,^26^].

Not only do histones express their toxicity through direct mechanisms, they are capable of initiating receptor-mediated processes that result in pro-inflammatory and cell-activating responses. Extracellular histones are recognized by PRRs as Toll-like receptors (TLRs), specifically TLR 2, TLR 4 and TLR 9, and nucleotide oligomerization domain (NOD)-like receptors, which results in expression of pro-inflammatory cytokines, inflammasome activation, and endothelial cell activation [27–29]. *In vitro* histone-treated endothelial cells showed increased expression of adhesion molecules ICAM-1, VCAM-1, e-selectin and P-selectin as well as tissue factor (TF) and von Willebrand factor (VWF) release, which contribute to platelet adhesion and initiation of the coagulation cascade [16^▪▪^,^29^–^33^]. *In vivo* studies in C57BL/6 mice have shown the release of VWF-containing Weibel Palade bodies, P-selectin, angiopoietin-2, interleukin (IL)-8, and endothelin-1, in response to administration of unfractionated histone (20 mg/kg) [7,16^▪▪^,^33^,^34^]. Collectively, there is ample evidence that extracellular histones cause endothelial dysfunction, which contributes to a procoagulant state and subsequent development of a thromboembolic event.

### Histone-mediated stasis of flow

Formation of an initial platelet plug on damaged endothelial cells causes a change in blood flow (Fig. 1b). Extracellular histones play a dual role in the induction of stasis. Firstly, histones can indirectly contribute to platelet activation and aggregation via endothelial damage as described in the previous paragraph. Secondly, platelets are activated by histones via direct binding in a charge-dependent manner leading to calcium influx [35]. Binding of histones to intra- and inter-cellular receptor activation, among which NOD-like receptors, TLR 2, 4 and 9, results in platelet activation [36]. In purified reaction systems, histones can induce platelet aggregation and increase the expression of P-selectin, phosphatidylserine, and FV/FVa [37,38]. A study by Lam *et al.*[39] did not observe platelet activation in platelet-rich plasma treated with histone H4 (2.5–10 μg/mL), as instead it was observed that histones are inhibited by plasma albumin, later confirmed by other studies [40,41]. The same experimental method using washed platelets did activate platelet aggregation [39]. These results emphasize the difficult interplay between factors involved in immunothrombosis and illustrate that the effects of histones in plasma may be different from those observed in experimental systems.

### Histone-mediated hypercoagulability

Extracellular histones affect both intrinsic and extrinsic coagulation pathways by their propensity to directly interact with coagulation proteins (Fig. 1c) [42]. Polyphosphate released by activated platelets is a potent coagulant that binds to FXII, initiating the intrinsic pathway [16^▪▪^]. Research by Huckriede *et al.* studying a cohort of SARS-COV-2 patients in whom extracellular histones were found to circulate, indicated the presence of a hypercoagulable state as shown by increased levels of thrombin:antithrombin. This rise is partially driven via the intrinsic pathway, as concluded from increased levels of multiple coagulation factors complexes as Kallikrein:C1 esterase inhibitor, FXIa:α1 antitrypsin, FXIa:antithrombin, and FIXa:antithrombin [5]. The extracellular histones released in these patients likely contribute to their hypercoagulable state [5,43].

The extrinsic coagulation pathway is initiated by TF binding to FVIIa. Histones play a role in the release of TF by activating the endothelium [16^▪▪^,^29^]. Additionally, *in vitro* experiments indicated the activation of FSAP by histones. Histone H2A, H3, and H4 were effective in promoting autoactivation of FSAP, with a half-maximal effect at 0.4 ug/ml of histones [44], a concentration that is well within the range observed in several disease states that are associated with thromboembolic disease [45].

In platelet-rich plasma, a dose-dependent thrombin generation response to stimulation by histones (0–160 μg/ml) was reported [37]. A possible explanation for this was given by Abrams *et al.* who showed the direct binding of prothrombin to histones. Upon formation of the histone-prothrombin-FXa complex, prothrombinase activity was observed, in the absence of the cofactor FVa [46]. Histones thereby appear to bypass the presence of the otherwise potent essential cofactor protein FVa.

Proteomics analysis of fibrin clots has indicated the presence of histones H3 and H4 in clot structures [47]. Emerging evidence suggests that histones play a significant role in modulating both fibrin fibre thickness and network density. Specifically linker histone H1 has been found to promote the formation of thicker fibrin fibres and a more porous clot structure, whereas the core histones H2A, H2B, H3 and H4 contribute to the generation of thinner fibres and a denser mesh network [48]. Consequently, these variations in clot architecture influence the kinetics of clot lysis, thereby impacting the degree to which thrombi cause clinical symptoms [49].

## INTERVENTIONS TO COUNTERACT THE THROMBOEMBOLIC EFFECTS OF HISTONES

Counteracting the pro-thrombotic effects of histones is a potential strategy to treat or prevent thromboembolic disease. Inhibition of histone activities can be obtained by neutralization through complex formation [with e.g. DNA, C-reactive protein (CRP), heparin or polyanions] or through proteolytic degradation of histones. Both methods, as detailed below, result in a complete or partial neutralization of the prothrombotic properties of histones.

### Neutralization of pro-thrombotic effects of extracellular histones by complex formation

Histones are extremely highly positively charged molecules, hence, negatively charged molecules are inherently suitable binding partners. Complexation of histones can prevent their binding to phospholipid membranes [25]. Additionally, complexed histones are hampered in their ability to bind to PRRs, thereby reducing their propensity to initiate pro-inflammatory and pro-coagulant events.

The most prominent, and physiological, binding partner of histones is DNA. A study by Marsman *et al.* showed that nucleosomes and histone-DNA complexes were not cytotoxic to HEK293 cells, whilst the free histones tested in the same assay were. After digestion of the DNA, histones became cytotoxic again, underscoring a protective role for DNA [50].

Other reported histone ligands are CRP and albumin [51]. Binding of CRP to histone H4 was assessed by ELISA, this complexation blocked ROS production induced by histone H4 in neutrophils, and diminished caspase 3 activity. These effects attenuate the pro-inflammatory and potentially pro-coagulant functions of histones [52]. Albumin appears to prevent histone-mediated platelet aggregation [33]. As albumin is one of the most prominent proteins in human blood, it is a good candidate to provide physiological protection from histone-induced activation processes.

Apart from hypothesized physiological neutralization, pharmacological interventions have been tested to diminish the cytotoxic and pro-coagulant effects of extracellular histones. Heparin is a negatively charged polysaccharide in use as an anticoagulant. Heparin provides protection against the pro-inflammatory and pro-coagulant functions of histones, independent of its anticoagulant properties [53]. A study of Wildhagen *et al.* proved *in vitro* binding of antithrombin affinity-depleted heparin to histone H3. This complexation completely neutralizes the cytotoxic effects of extracellular histones to endothelial cells [54]. Addition of unfractionated heparin (UFH) or low molecular weight heparin (LMWH) effectively neutralizes the cytotoxic effects of histones, reduces expression of pro-inflammatory cytokine levels, and inhibits heparinase activity [54–56]. A phase I clinical trial with low-coagulant heparin was successfully concluded in 2024 in critically ill sepsis patients [57].

Suramin, another polyanionic drug was studied in the context of extracellular histones neutralization and prevention of endothelial dysfunction [58]. Suramin was shown to have an overall anti-inflammatory effect *in vitro* and reduced thrombin in recalcified pooled healthy human plasma. However, Suramin was not able to neutralize the effect of citrullinated histones, which are released during NETosis [58].

### Neutralization of histones by proteolytic cleavage

Proteolytic histone cleavage mitigates the cytotoxic and prothrombotic characteristics of extracellular histones [14,59,60^▪^]. *In vivo* confirmation thereof was provided by the presence of cleaved histones in SARS-COV-2 patients that associated with a lower amount of thromboembolic events [59]. Although details of this form of neutralization are still being studied, it is hypothesized that histone fragments have reduced activities compared to full-length histones in the many ways through which histones express their activation and toxic activities.

Proteases should share a similar localization as extranuclear histones to be of *in vivo* relevance to proteolytic regulation. Therefore, we here describe proteases released by neutrophils and circulating proteases that are studied in the context of histone proteolysis (Table 1).

**Table 1:** Characteristics of proteases able to cleave histones. The origin, type of protease, and confirmed cleavage site on histone subtype(s)

Protease	Origin	Type of protease	Confirmed cleavage site on histone	Reference
Neutrophil elastase	Neutrophil	Serine	Thr32 (histone H3)	[65]
Cathepsin G	Neutrophil	Serine	Leu 20 (histone H3)	[65]
Proteinase 3	Neutrophil	Serine	Lys23 & Thr32 (histone H3)	[65]
APC	Liver	Serine	Arg26-Lys27 (histone H3)	[60^▪^]
FSAP	Hepatocytes	Serine	Multiple cleavage sites (Histone H1, H2A, H2B, H3, H4)	[50]
MMP	Various cells	Zinc-dependent	N-terminal cleavage (histone H3)	[77]

#### Histone cleavage by proteases released from neutrophils

Azurophilic granules are shed from neutrophils during NETosis, releasing three serine proteases: neutrophil elastase (NE), Cathepsin G, and Proteinase 3. Although essential for antimicrobial defence, these proteases possess the capacity to cleave many substrates, including histones [61].

Upon neutrophil activation, NE is released along with histones, both being NET-bound [61,62]. A positive correlation has been found between plasma NE levels and extracellular histone H3 concentrations in COVID-19 patients [63]. The simultaneous exposure to the bloodstream of histones and their potential down-regulator, NE, might represent a likely regulatory pathway whereby the activator (histones) is made available simultaneously with its down-regulator (NE). Co-incubation of human histone H3 with NE demonstrate histone H3 degradation, as evidenced by immunoblotting [64] and mass spectrometry [65]. NE in itself can cleave several coagulation and fibrinolytic proteins [66], however, the relevance of *in vivo* histone cleavage by NE still has to be further investigated.

Release of cathepsin G from neutrophils in proximity to histones during NETosis suggests that there could be an *in vivo* role for cathepsin G in histone proteolysis. Samples of recombinant nucleosomes incubated with cathepsin G were analysed by immunoblotting and cleaved histone H3 was observed [65,67–69]. Cathepsin G has been studied in the context of coagulation. FVIII can be cleaved by cathepsin G, however, this cleaved form can still be activated by thrombin [70]. The role of each protease on the coagulation independent of histones proves that there is a complex mechanism behind the term immunothrombosis.

A third protease in the azurophilic granules of neutrophils is proteinase 3. *In vitro* assays demonstrated the ability of proteinase 3 to cleave the N-terminal region of histone H3, as confirmed by mass spectrometry analysis. Depletion of proteinase 3, along with other serine proteases, in cells resulted in the abolishment of histone H3 cleavage, underscoring a role of proteinase 3 in this process [65]. Proteinase 3 might not only have an anticoagulant effect by histone cleavage, as it is able to cleave phosphatidylserine, a pivotal molecule of coagulation processes [71].

#### Histone cleavage by proteases circulating in the bloodstream

Histones can not only be proteolysed when bound to NETs, once released in the bloodstream, they can encounter plasma proteases that can degrade them. Three proteases that are present in the circulation are candidates to cleave extracellular histones *in vivo.*

##### Activated protein C

Activated protein C (APC) is the central protease in the anticoagulant protein C pathway. The natural substrates of APC are coagulation cofactors FVa and FVIIIa and their precursors, but APC also has reported anti-inflammatory properties [72]. Protein C deficiency results in a severe prothrombotic phenotype [73]. Given that APC can effectively target extracellular histones, it cannot be excluded that failure to downregulate the prothrombotic properties of histones contributes to the thromboembolic manifestations seen in individuals with circulating histones [60^▪^]. *In vitro* studies have shown the ability of APC to proteolyse histone H3. Molecular docking analyses combined with protein engineering studies have revealed specific residues, including Arg26 and Lys27, in histone H3 can align with the substrate binding pocket of APC, which supports the capacity of APC to cleave histone H3 [60^▪^]. Additionally, the derived results provided a proof-of-concept for the development of novel proteases (e.g., APC variants) that can specifically reduce the cytotoxicity of histones.

##### Factor VII-activating protease

Another coagulation protease is Factor VII-activating protease (FSAP), which circulates in an active and inactive form in human plasma. Recent data suggest that FSAP activation is closely linked to inflammatory processes [74]. Extracellular histones are known to trigger FSAP activation, and activated FSAP can in turn proteolytically cleave histones [44]. This was tested using *in vitro* experiments involving the incubation of plasma-purified FSAP with histones. Proteolytic cleavage of all histone subtypes at multiple cleavage sites was shown and cleavage reduced histone mediated cytotoxicity. Conversely, experiments using FSAP-depleted serum fail to demonstrate a cytoprotective function [50,75]. The significance of FSAP-mediated cleavage of histones for thrombosis remains unknown.

##### Matrix metalloprotease

Matrix metalloproteases (MMPs) are a diverse group of zinc-dependent proteases with primary roles in tissue remodelling, wound healing, and inflammation [76]. Observations of histone H3 N-terminal tail cleavage during eukaryotic development hint at a role for MMPs in histone cleavage. *In vitro* cell culture and Western Blot analysis have demonstrated MMP-2 to cleave histone H3 [77,78]. Also, MMP-9-mediated cleavage of histone H3 plays a role in osteoclast formation and melanoma cells. Intracellular function of MMPs can play a role in the extracellular space upon release in the bloodstream [79,80]. However, their exact role should be investigated further.

## CONCLUSION

Histones, essential nuclear proteins governing chromatin structure and epigenetic regulation, possess significant pro-inflammatory and pro-coagulant attributes upon release to the extracellular milieu. Whenever histones enter the circulation, this may instigate endothelial dysfunction, promote clot formation, and reinforce clot stability. Such imbalance, if not promptly and properly counterbalanced may impact the clinical status of patients with circulating extracellular histones. Strategies aimed at histone neutralization, either through histone complexation or histone proteolysis, offer potential avenues for ameliorating histone-induced cytotoxicity and prothrombotic tendencies, such as are seen in immunothrombotic or thrombo-inflammatory patients. However, despite current research efforts, our understanding of the intricate pathways involved in the exposure of histones, their neutralization and clearance from circulation, and the ways these affect thrombotic disease, remains incomplete. At present few hypothetical models can be proposed, following which histones act as prothrombotic agents. Complexed or proteolysed histones lose the ability to bind to phospholipid membranes as well as the ability to be recognized by PRRs and consequently can no longer activate endothelium, platelets and interact with coagulation proteins; collectively diminishing the overall thromboembolic state. Leveraging our understanding of the mechanisms behind complexation and proteolysis of histones may pave the way for the development of novel pharmacological interventions aimed at alleviating or prevention of the complications observed in patients in whom extracellular histones are known to circulate and contribute to their overall clinical status.

## Acknowledgements

*Support by the Cardiovascular Research Institute Maastricht, CARIM, to ongoing projects in histone-mediated disease is highly appreciated. GAFN and KW receive funding from the EU (PRAETORIAN Doctoral Network) and Novo Nordisk to study histone-mediated pathologies.*

### Financial support and sponsorship

*None.*

### Conflicts of interest

*G.A.F.N. and K.W. are inventors of a patent held by the Maastricht University on histone neutralization (WO2019122127A1). G.A.F.N, is advisor and shareholder of Matisse Pharmaceuticals B.V. a company that holds a license to a patent of the Maastricht University on the use of nonanticoagulant heparin the treatment of inflammatory diseases and sepsis (US9155756B2).*

## REFERENCES AND RECOMMENDED READING

Papers of particular interest, published within the annual period of review, have been highlighted as:

▪ of special interest
▪▪ of outstanding interest
